# Performance Evaluation of the MyT4 Technology for Determining ART Eligibility

**DOI:** 10.1371/journal.pone.0165163

**Published:** 2016-10-25

**Authors:** Nádia Sitoe, Rosa Macamo, Bindiya Meggi, Ocean Tobaiwa, Osvaldo Loquiha, Timothy Bollinger, Lara Vojnov, Ilesh Jani

**Affiliations:** 1 Instituto Nacional de Saúde, Maputo, Mozambique; 2 Clinton Health Access Initiative, Maputo, Mozambique; University of Pittsburgh, UNITED STATES

## Abstract

**Background:**

In resource-limited countries, CD4 T-cell (CD4) testing continues to be used for determining antiretroviral therapy (ART) initiation eligibility and opportunistic infection monitoring. To support expanded access to CD4 testing, simple and robust technologies are necessary. We conducted this study to evaluate the performance of a new Point-of-Care (POC) CD4 technology, the MyT4, compared to conventional laboratory CD4 testing.

**Methods:**

EDTA venous blood from 200 HIV-positive patients was tested in the laboratory using the MyT4 and BD FACSCalibur^™^.

**Results:**

The MyT4 had an r^2^ of 0.82 and a mean bias of 12.3 cells/μl. The MyT4 had total misclassifications of 14.7% and 8.8% when analyzed using ART eligibility thresholds of 350 and 500 cells/μl, respectively.

**Conclusions:**

We conclude that the MyT4 performed well in classifying patients using the current ART initiation eligibility thresholds in Mozambique when compared to the conventional CD4 technology.

## Introduction

Globally, approximately 17 million HIV-positive individuals are on antiretroviral therapy (ART). This currently falls above the UNAIDS target of providing ART to 15 million people by 2015 [1]. Furthermore, according to the 2016 World Health Organization (WHO) Consolidated ART guidelines, approximately 37 million HIV-positive individuals are eligible for ART [2].

The 2016 WHO Consolidated ART guidelines recommend that ART should be initiated in all adults living with HIV, regardless of WHO clinical stage and at any CD4 cell count. These guidelines strongly recommend using viral load testing instead of CD4 testing to monitor ART. Viral load testing is important to diagnose and confirm treatment failure [2]. These new HIV treatment guidelines result in a less central role for CD4 testing in the management of HIV infection. It is therefore to be expected that CD4 testing will be phased out as a tool to assess eligibility of HIV+ patients for ART in the future. However, CD4 testing may remain a valuable tool for managing opportunistic infections [3].

Despite these recent recommendations, in Mozambique and similar high burden resource-limited countries, CD4 testing remains critical for determining ART eligibility for HIV-positive individuals [4] as well as monitoring patients on ART since viral load testing is largely unavailable. Additionally, CD4 testing is the best predictor of disease progression [5–8] and superior to symptomatic staging [9–11]. Due to limited resources in developing high burden HIV countries, programs prioritize patients to receive ART based on disease progression and illness.

In Mozambique, over 500,000 ART eligible HIV-positive individuals are not on ART and less than 25% of health care facilities with ART services have a CD4 technology on-site. Generally, CD4 testing is performed at centralized laboratories. Patients who attend health care facilities lacking on-site CD4 testing do not have reliable access to testing and often require several health care facility visits for drawing blood and receiving test results. Within the conventional CD4 referral network, CD4 samples often require transportation over long distances and difficult roads, with transportation being expensive and often not available. Moreover, specimens for CD4 testing have a short period of stability and do not stand transportation at tropical room temperature. Unfortunately, the cost and time required to build new laboratories is unaffordable to decentralize CD4 and other tests.

Point-of-Care (POC) testing has been found to reduce test turnaround times and the subsequent loss to follow up observed in the conventional CD4 referral network [12, 13]. The simplicity, rapid results, and lack of infrastructure requirements of POC technologies allows for significant decentralization of the CD4 testing network beyond reference laboratories [14]. Previously available POC CD4 technologies have been successfully technically evaluated [15–17] and showed significant positive patient impact [12, 13]. For optimal utilization and reliable testing, it is critical to monitor such a decentralized network to ensure consistently high quality testing.

Currently the Alere Pima and BD FACSPresto POC CD4 technologies are the only technologies available with European Union Conformity (CE-IVD) regulatory approval for in vitro diagnostic use and WHO prequalification status. Additional technologies, such as the MyT4, are in the product pipeline [14]. We, therefore, assessed the technical performance of a newly developed CD4 technology, the MyT4, to provide accurate CD4 test results.

## Materials and Methods

### Study Population

Samples from routine clinic referrals were collected in March and April 2014. Samples from two hundred HIV-positive patients and attending two health care facilities in Maputo City (Hospital de Polana Caniço and Hospital Central de Maputo) were included in the study analysis. Children above five years of age, adolescents, and adults currently require a CD4 test to determine ART eligibility in Mozambique and thus were included in this study. This study was reviewed and approved by the National Bioethics Committee for health in Mozambique (Approval number 318/CNBS/12). Informed consent was not sought from participants, as the samples used were remnants from other requisitioned routine CD4 testing conducted in the laboratory. Additionally, in each standard testing requisition form, patient information, such as age and gender, were collected.

### Study Design

This independent cross-sectional technical methods comparison study compared the performance of the MyT4 CD4 technology (developer and manufacturer: Zyomyx, Inc. Fremont, CA, USA; distributor: Mylan Laboratories, Ltd., Canonsburg, PA, USA) with conventional CD4 testing of the BD FACSCalibur^™^ (Becton Dickinson, East Rutherford, NJ, USA). Trained laboratory technicians for each platform were blinded and performed all testing using venous blood from EDTA-coated tubes according to the manufacturers’ instructions at the Instituto Nacional de Saúde. Demographic data and test results from each patient were collected and entered into a Microsoft Excel database. All CD4 tests were consecutively processed on the same day as sample extraction. The physician was only provided with the CD4 result from the BD FACSCalibur^™^.

### CD4+ T-Cell Counts

The samples were collected by venipuncture into K_3_EDTA vacuum tubes (Becton Dickinson, USA). The enumeration of CD4+ T-cells was first performed by single-platform flow cytometry technology, BD FACSCalibur^™^, on fresh whole venous blood within six hours of collection. On the BD FACSCalibur^™^ flow cytometer, the CD3^FITC^/CD8^PE^/CD45^PerCP^/CD4^APC^ MultiTEST reagents, TruCOUNT tubes, and MultiSET software were used in a lyse-no-wash protocol to determine absolute and percent values for T-cell subsets. The same samples were tested using the point-of-care (POC) device MyT4. This POC instrument is semi-automated, has dried reagents, and produces results within 15 minutes. In this POC assay, the EDTA blood sample was introduced in the reaction chamber of the test cartridge that contained a zyosphere reagent pellet with heavy CD4-specific particles and magnetic CD14-specific particles using a capillary tube with 100μl of total blood. A sedimentation stage follows, during which the heavy CD4^+^ T-cells stack in the tube inside the bottom of the test cartridge. The stack height is then read visually using a built-in lens with a calibrated ruler [17, 18].

### Quality Control

Daily quality controls were run on the BD FACSCalibur^™^ using the TruCount beads, per manufacturer’s instructions. For the MyT4, each test cartridge has a control window to be interpreted by the end-user to validate each test result. The conventional CD4 technology used in the evaluation successfully participates in the Quality Assurance Systems International (QASI) external quality assurance (EQA) program and the national EQA program for CD4 testing (PNAEQ). All laboratory technologists performing the conventional CD4 technologies included in this study are trained annually in good laboratory practice, immunophenotyping for flow cytometry, and biosafety. The manufacturer trained the MyT4 operators. Results were matched after testing was completed using an internal laboratory code.

### Statistical Analysis Methods

The linear CD4 cell count range of the MyT4 was equal to or above 10 cells/μl and equal to or below 950 cells/μl. All results from the comparator method that were outside of this linear range were omitted from linear regression and Bland-Altman analyses. The technical performance characteristics of the MyT4 were analyzed using standard statistical methods for evaluating diagnostic technologies. Bland-Altman analyses were performed to determine the bias and 95% limits of agreement between the MyT4 and BD FACSCalibur^™^ [19]. The y-axis on each Bland-Altman plot highlights the difference between the two methods (test technology—gold standard). The absolute CD4 counts from the MyT4 in the range ≥10 cells to ≤950 cells/μl were directly compared to those from the BD FACSCalibur^™^ conventional CD4 technology using linear regression analysis and calculating the coefficient of determination (r^2^). The percentage similarity was calculated as previously described [20]. Just before the study period, the WHO increased the recommended ART initiation eligibility threshold from 350 cells/μl [21] to 500 cells/μl [4]. The misclassification of the MyT4 was calculated compared with the BD FACSCalibur^™^ using thresholds of 350 cells/μl and 500 cells/μl. Misclassification was defined using the equations below:

Upward misclassification percentage: the # of patients incorrectly categorized above the threshold by the MyT4 divided by the # of patients categorized below the threshold by the BD FACSCalibur^™^.

Downward misclassification percentage: the # of patients incorrectly categorized below the threshold by the MyT4 divided by the # of patients identified as above the threshold by BD FACSCalibur^™^.

The misclassification analyses included all observations, including those outside of the MyT4 range.

Finally, repeatability was calculated from 39-paired samples tested on the same instrument by the same technician for the MyT4 to determine the coefficient of variation. The samples used for repeatability were in the range of 13 to 1915 cells/μL as measured by the FACSCalibur. All specimens received in the laboratory during the last four days of the study were utilized for this analysis. Specimens included some with low CD4 counts (less than 350 cells/μl, N = 12), some with medium CD4 counts (less than 500 cells/μl, N = 8) and some with high CD4 counts (more than 500 cells/ul, N = 19).

All statistical analyses were performed with GraphPad Prism^™^, R and/or Microsoft Excel.

## Results

This evaluation used two hundred samples of venipuncture-derived EDTA blood collected in routine services from HIV-positive patients. The majority (143) of included patients were over 18 years of age. Approximately 50% of the samples were from female patients. The median age of patients was 32 years (range: 5–78 years) (Table 1). The median CD4 count according to the BD FACSCalibur^™^ was 386 cells/μl (range: 8–923 cells/μl), while the median CD4 count according to the MyT4 was 400 cells/μl (range: 25–925 cells/μl).

**Table 1:** Patient sample demographics.

	Female		Male	
**Median age (IQR)**	32 (14–41)		32.5 (15–43)	
**CD4 count**	**MyT4**	**FACSCalibur**	**MyT4**	**FACSCalibur**
**<100**	6	8	9	11
**100–349**	23	29	27	28
**350–499**	24	15	24	23
**500–949**	37	38	29	28
**≥ 950**	18	18	3	2
**Total**	108 (54%)		92 (46%)	

**Fig 1:**
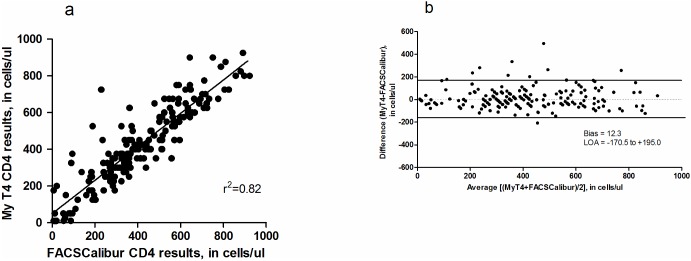
Regression plot (a) and Bland-Altman difference plot (b) for comparison of the MyT4 with the BD FACSCalibur^™^. (a) In the Bland-Altman difference plot the vertical axis is the difference between the CD4 counts from the two methods while the horizontal axis is the average CD4 count from each patient.

Because the linear range of the MyT4 test is between 10–950 cells/μl, samples were excluded if less than 10 cells/μl or greater than 950 cells/μl. Compared with the BD FACSCalibur^™^, the MyT4 had a coefficient of determination, r^2^, of 0.82 and mean bias of 12.3 cells/μl (95% LOA: -170.5–195.0 cells/μl) (Fig 1a and 1b). The mean percentage similarity was 105.4% (39.3% SD).

The total misclassification rates were below 18% for each of the analyzed thresholds (Table 2). Upward misclassification rates were determined as the number of patients incorrectly categorized above the threshold by the MyT4 over the number of patients categorized below the threshold by the BD FACSCalibur^™^. Downward misclassification rates were determined as the number of patients incorrectly categorized below the threshold by the MyT4 over the number of patients categorized above the threshold by the BD FACSCalibur^™^. At the ART initiation eligibility threshold of 350 cells/μl, the upward misclassification rate was 17.4% (95% CI: 9.3–28.4%), while the downward misclassification rate was 12.8% (95% CI: 6.9–20.8%). At the ART initiation eligibility threshold of 500 cells/μl, the upward misclassification rate was 5.7% (95% CI: 2.1–11.9%), while the downward misclassification rate was 13.9% (95% CI: 6.5–24.7%).

**Table 2:** Misclassification and agreement statistics.

	Misclassification				Agreement		
Threshold	Upward	Downward	Total (95% CI)	Total (N)	Upward	Downward	Total
	(95% CI)	(95% CI)			(95% CI)	(95% CI)	(N)
350	15.6%	10.60%	12.50%	25	89.80%	83.90%	175
	(8.3–25.6)	(5.8–17.4)	(8.3–18.4)		(85.8–93.8)	(776–90.1)	
500	53%	10.40%	7.50%	15	91.10%	93.50%	185
	(2.0–11.1)	(4.9–18.9)	(4.3–13.7)		(86.7–95.6)	(90.23–96.8)	
**Total**				43			157

When children were excluded, an analysis of only adult patients (≥ 18 years of age) produced similar results to the larger data set. The mean bias was 12.4 cells/μl (95% LOA: -170.12–194.92) and the r^2^ was 0.77. A sub- analysis of results from only children (5–18 years of age) was also similar to the larger data set. The mean bias was 17.7 cells/μl (95% LOA: -182.22–217.62) and the r^2^ was 0.77.

Finally, performing two independent tests using the same device by the same operator in the laboratory from 39 patients produced a coefficient of variation for the MyT4 of 5.4% (95% CI: 0.1–10.7%).

## Discussion

The main aim of this study was to compare the MyT4 CD4 technology to the BD FACSCalibur^™^ for both the 2010 and 2013 WHO ART initiation eligibility thresholds of 350 and 500 cells/μl, respectively. Sensitivity to identify patients eligible for ART initiation was good. The overall mean bias was 12.21 cells/μl and the total misclassification rates across all two tested thresholds were below 18%. Although the MyT4 produced higher counts in comparison to BD FACSCalibur^™^, the differences were small and results were within acceptable ranges. Currently, there are not clear standards that have been set for technical performance of CD4 testing when developing technologies. Diagnostic regulatory bodies usually decide the acceptable ranges of variation when make decisions about technology incorporation. In our past experience with POC CD4 assays evaluation, the PIMA CD4 technology had a bias of -62.3 cells/μl and a misclassification rate of 14.8% [22], values similar to those observed with the MyT4 assay. These results were comparable to previous POC CD4 technologies evaluations currently being used in developing countries [16, 22, 23].

Implementing accurate POC CD4 technologies will further decentralize the CD4 testing network, particularly to remote health care facilities as well as health care facilities lacking reliable access to centralized CD4 testing [24]. POC CD4 technologies can improve quality access to CD4 testing and subsequent HIV monitoring and staging. Although viral load is an important tool for HIV monitoring and the 2016 WHO Consolidated ART guidelines suggest the phasing out of CD4 testing as a tool to assess ART eligibility, the use of CD4 testing in limited resource countries may still be used to prioritize patients in most need ART initiation, to monitor disease progression and opportunistic infections [3]. Furthermore, in countries with financial constraints, viral load testing may be too expensive to be immediately available to all HIV-positive patients. In Mozambique, the viral load testing will be prioritized to HIV infected children and pregnant women from areas with high HIV prevalence. For these reasons, a simple, cheap and robust POC CD4 technology such as the MyT4 may be necessary and useful.

One of the limitations of the MyT4 technology is the narrow linear range within the lower and upper measurable limit of 10–950 cells/μl compromising CD4 quantification results above or below these limits. The impact of this limitation on ART initiation was minimal when focusing on the primary thresholds (350 and 500 cells/μl) of interest. Furthermore, CD4 T-cell counts of HIV-infected children under the age of five years old are often above 1,000 cell/μl, therefore the use of MyT4 for CD4 enumeration within this critical population would not be as useful. For this population, HIV staging and monitoring is typically done using CD4 percent testing.

Despite the comparable performance of the MyT4, the use of EDTA patient blood by laboratory technicians instead of using finger prick blood was a limitation. Although samples were not tested in the intended setting of lower level health care facilities by nurses, other studies observed that the performance by either laboratory technicians or nurses using POC CD4 technologies was similar [16, 17, 22, 23]. A recent study of the MyT4 evaluation in the intended field setting using finger-prick blood also highlighted comparable performance to conventional technologies [17]. In the same study, Mwau *et al*. observed that formal microscopy skills were not necessary for successful testing and result interpretation [17]. Further studies would be critical to understand the performance of the MyT4 technology in the intended field setting by the intended end users. Additionally, the sample size of the study was small; however, the 95% confidence intervals of the misclassification rates at the 350 and 500 cells/μl ART initiation eligibility thresholds were within +/- 10%.

When selecting and implementing POC CD4 technologies, several factors are important to consider. Thoughtful technology selection and placement, robust supply chain, training, monitoring, and a quality assurance plan will help ensure a successful program. This study confirmed that the MyT4 technology performs comparably to conventional laboratory technologies and can be used within the HIV testing system to allow improved patient health care and increased access to quality services. Future studies will be imperative to better understand the potential acceptability and feasibility of the technology and potentially useful external quality assurance schemes.
